# Current Breastfeeding Attitudes, Knowledge and Confidence of Obstetricians and Gynaecologists in Australia and New Zealand

**DOI:** 10.1111/ajo.70055

**Published:** 2025-07-22

**Authors:** Gabrielle Cher, Diana M. Bond, Natasha Nassar, Katy Hunt, Jane Svensson, Olga Aleshin, Antonia Shand

**Affiliations:** ^1^ Royal Hospital for Women Randwick New South Wales Australia; ^2^ Faculty of Medicine and Health, University of New South Wales Sydney Australia; ^3^ Child Population and Translational Health Research Children's Hospital at Westmead Clinical School, Faculty of Medicine and Health, the University of Sydney Sydney New South Wales Australia; ^4^ Leeder Centre for Health Policy, Economics, and Data Faculty of Medicine and Health, the University of Sydney Sydney New South Wales Australia

**Keywords:** breastfeeding attitudes, breastfeeding knowledge, lactation, obstetricians and Gynaecologists, physicians

## Abstract

**Background:**

There are limited data on what obstetricians and gynaecologists (O&G) know and think about supporting breastfeeding women.

**Aims:**

To investigate breastfeeding attitudes, knowledge and confidence of Australian and New Zealand O&G specialists and trainees in educating, assessing and managing breastfeeding women.

**Materials and Methods:**

An online REDCap survey was distributed via email in February 2023 to fellows and trainees of the Royal Australian New Zealand College of Obstetricians and Gynaecologists (RANZCOG). The survey included questions on demographic characteristics, knowledge, attitudes and confidence about breastfeeding.

**Results:**

Of 312 (11%) respondents, 63% were > 40 years old, 78% female and two‐thirds had personally breastfed. Half had no formal breastfeeding education. Mean score related to attitude was 4.8/7 (71.1%) with higher scores associated with extra training (β = 0.44 (95% CI 0.04, 0.84)) and personal breastfeeding (β = 0.37 (95% CI 0.15, 0.60)). Mean correctly answered knowledge score was 9/12 (75%). After adjusting for covariates, the main factors associated with higher knowledge were personal breastfeeding (β = 0.45 (95% CI 0.21, 0.69)) and being female (β = 0.58 (95% CI 0.10, 1.07)). Overall mean confidence score was 4.8/7 (68.6%); however, only 37% felt confident in managing breastfeeding challenges, and 60% would value more breastfeeding education. Factors associated with increased confidence included personal breastfeeding (β = 0.52 (95% CI 0.31, 0.73)), increased age (β = 0.39 (95% CI 0.64, 0.71)), and extra training (β = 0.84 (95% CI 0.46, 1.21)).

**Conclusions:**

Confidence about breastfeeding in RANZCOG specialists and trainees was low. The majority of respondents wanted more formal and improved breastfeeding education and training. Breastfeeding educational resources and ongoing training should be developed for O&G trainees and specialists.

## 1 Introduction

Breastfeeding is well established in protecting infant and maternal health and stands as a critical public health matter [1, 2, 3]. Breastfeeding mothers' need education and support to breastfeed successfully [4]. Yet despite its well‐established benefits, only 39% of Australian mothers exclusively breastfeed to 3 months and 15% reach 5 months [5], which is less than the recommended duration of 6 months of exclusive breastfeeding [6]. The most cited reasons for not continuing breastfeeding in the Australian National Infant Feeding Survey (2010) were breastfeeding‐related challenges [5] that can be addressed and managed by trained professionals.

The UNICEF Baby Friendly Initiative states that medical staff should have adequate knowledge, competence and skills to support breastfeeding and be provided the necessary training to do so [7]. Moreover, the American College of Obstetrics and Gynaecologists advises that obstetric care providers develop and maintain knowledge and skills in guiding, assessing and supporting breastfeeding mothers [2]. However, studies have found a lack of knowledge and confidence among different medical professional groups in supporting, assessing and managing breastfeeding women [8, 9]. A study in the United Kingdom found that medical schools were not adequately preparing students for breastfeeding assessment and knowledge and that 93% of medical students requested further breastfeeding education [10]. Dovetailing this, a 2022 Cochrane review indicated that doctors were not receiving adequate training to support and manage breastfeeding challenges [11]. In Canada, breastfeeding knowledge was investigated among paediatric trainees and found that most junior doctors lack the knowledge and training to manage breastfeeding difficulties [12]. Other American and Canadian studies demonstrate inadequate breastfeeding knowledge and standards among doctors providing obstetric care [13, 14]. In Australia, a study of General Practitioners found that nearly all the participants lacked formal breastfeeding training, and despite having positive attitudes towards breastfeeding, their knowledge and confidence in providing support and care to breastfeeding mothers was suboptimal [15]. Brodribb et al. [16] found that nearly 80% of Australian General Practitioners felt that they needed further breastfeeding education.

Obstetricians and gynaecologists have a critical role in maternity care, as breastfeeding advocates, educators and supporters. Evidence shows that implementing interventions to support breastfeeding can increase the rate, exclusivity and duration of breastfeeding [17]. O&G trainees and specialists, therefore, need knowledge and training to provide evidence‐based and appropriate care to mothers. Instituting formal breastfeeding education for physicians has been shown to not only improve breastfeeding knowledge among doctors but also ensure better patient outcomes [18, 19]. However, it is not known if formal breastfeeding education is currently provided to O&G doctors working in Australia and New Zealand and whether this impacts their confidence and ability to educate and manage breastfeeding women. This study aimed to investigate breastfeeding knowledge and attitudes about breastfeeding, and confidence in providing breastfeeding care and support to breastfeeding women among O&G doctors in Australia and New Zealand.

## 2 Materials and Methods

### 2.1 Data Collection

A link to an anonymous Research Electronic Data Capture (REDCap) questionnaire was distributed by email in February 2023 to specialist trainees, fellows and subspecialists of the Royal Australian New Zealand College of Obstetricians and Gynaecologists (RANZCOG) in Australia and New Zealand by the RANZCOG. Study data were collected and managed using REDCap electronic data capture tools hosted at the University of Sydney. REDCap is a secure, web‐based software platform designed to support data capture for research studies [20]. A single reminder email was sent after 2 weeks. The study investigators did not have access to the email addresses of the responders. RANZCOG trainees and specialists who stated that they were providing antenatal and postnatal care were included in this study.

The survey included four sections: Attitudes and beliefs regarding breastfeeding, knowledge of breastfeeding, confidence in providing breastfeeding support and demographic information (Appendix 1). There were nine questions in Section 1 regarding attitudes and beliefs and responses were on a 5‐point Likert‐type scale (strongly disagree, disagree, neither agree nor disagree, agree, strongly agree). The 12 knowledge‐based questions in Section 2 covered breastfeeding physiology and basic sciences, benefits of breastfeeding, common breastfeeding issues and maternal conditions and their effect on breastfeeding. The questions included were developed from the literature review conducted in the process of this research project. Responses for the knowledge section were on a 4‐point Likert‐type scale (disagree, neither agree nor disagree, agree and don't know). Section 3 contained eight confidence‐based questions similarly using the 5‐point Likert‐type scale as Section 1. Negatively worded items were reverse scored. Higher scores indicated more positive attitudes in Section 1, correct answers in Section 2, and a higher level of confidence in Section 3. Section 4 contained demographic information, including whether formal breastfeeding education or training had been received during medical school or as part of mandatory hospital training. Breastfeeding history was determined by the participant as: (1) those that had personally breastfed themselves; (2) having a partner who had breastfed; or (3) never breastfed or been a partner to a breastfeeding woman. There was also a free text box at the conclusion of the survey to include any additional comments the participant wished to add.

The survey was based on previous surveys and questions developed by midwives, obstetricians and researchers and was piloted by lactation consultants, midwives and doctors who care for breastfeeding mothers. The pilot survey took approximately 3–6 min to complete. Ethics approval was obtained from the South Eastern Sydney Local Health District Human Research Ethics Committee (ETH12255).

### 2.2 Statistical Analysis

Frequency and percentage tabulation of the demographic characteristics of survey participants were calculated using descriptive statistics. Mean scores for knowledge, attitudes and behaviours were compared across demographic characteristics using independent sample *t*‐test analyses and ANOVA. Univariate and multivariate regression analyses were used to assess association between main factors of interest and each outcome of attitude, knowledge and confidence scores. Only factors with *p* value < 0.2 on univariate analyses were then included in the multivariate analyses to assess the overall impact on outcomes. Data were analysed using SPSS Version 28 (IBM SPSS Statistics, 2021 IBM Corp., Armonk, NY, USA) and *p* value < 0.05 was considered statistically significant.

Comments related to open‐ended questions were thematically analysed by two researchers who independently coded the data into emergent themes using the study objectives. Themes were then clustered into overarching themes, compared and discussed with a third researcher until consensus was reached. Quotations directly relating to the main themes were identified.

## 3 Results

A total of 326 participants responded, with 312 completed surveys, yielding an estimated response rate of 11% [21]. The majority of the participants were above 40 years of age (63%), specialists (75%) and female (78%). Fifty per cent of participants had no formal breastfeeding education or training, and 81% had a breastfeeding history, having either personally breastfed (63%) or had a breastfeeding partner (18%). Demographic characteristics of the survey participants are summarised in Table 1.

**TABLE 1:** Demographic characteristics of the participants.

Demographic characteristics	Number	%
Age Group (years) (*n* = 302)		
20–39	109	36.1
40–59	147	48.7
≥ 60	46	15.2
Sex (*n* = 307)		
Female	241	78.5
Male	66	21.5
Country of medical training (*n* = 312)		
Australia/New Zealand	260	83.3
Other	52	16.7
Trainee or FRANZCOG/Specialist (*n* = 312)		
RANZCOG trainee	79	25.3
FRANZCOG/Specialist	233	74.7
Years in practice for specialist (years) (*n* = 233)		
Less than 5	62	26.6
5–10	40	17.2
More than 10	131	56.2
Type of role (*n* = 312)		
Obstetrics	66	21.2
Obstetrics and Gynaecology	216	69.2
Gynaecology	21	6.7
Other	9	2.9
Formal breastfeeding education or training (*n* = 312)		
Yes	150	48.1
No	155	49.7
Don't know	7	2.2
Have sought additional training about breastfeeding (*n* = 312)		
Yes	66	21.2
No	246	78.8
Breastfeeding history (*n* = 312)		
Personally breastfed	193	62.2
Partner has breastfed	57	18.1
Never breastfed or had breastfeeding partner	55	17.5
Prefer not to say	7	2.2
Accredited Baby‐Friendly Health Initiative facility (*n* = 312)		
Yes	257	82.3
No	20	6.4
Missing	35	11.2

Attitudes and behaviours towards breastfeeding are presented in Table 2. Attitudes towards breastfeeding were largely positive, with an overall mean attitude score of 6.4/9 (71.1%). Around two‐thirds (70%) disagreed with the statement that breastfeeding problems are the domain of a midwife or lactation consultant. One‐third (33%) were neutral about whether a mother should be encouraged to use formula if struggling to breastfeed.

**TABLE 2:** Confidence and attitudes and beliefs about breastfeeding support and care.

Confidence (*n* = 314)	Agree	Neutral	Disagree
I play a role in breastfeeding promotion and education for my patients	262 (83.4%)	28 (8.9%)	24 (7.6%)
I help facilitate immediate and uninterrupted skin‐to‐skin contact between newborn infants and mothers at all births	279 (88.9%)	20 (6.4%)	15 (4.8%)
I support mothers in initiating and maintaining breastfeeding	272 (86.6%)	34 (10.8%)	8 (2.5%)
I believe obstetricians should counsel mothers on the use and risks of feeding bottles, teats and pacifiers	96 (30.6%)	120 (38.2%)	98 (31.2%)
I can identify if an infant is sucking/feeding well at the breast	175 (55.7%)	56 (17.8%)	83 (26.4%)
I feel confident in managing breastfeeding‐related challenges	115 (36.6%)	78 (24.8%)	121 (38.5%)
I am aware of the available services to refer my patients to for breastfeeding support and care	301 (95.9%)	7 (2.2%)	6 (1.9%)
I would value more formal breastfeeding education or training	188 (59.9%)	64 (20.4%)	62 (19.7%)
Attitudes and Beliefs about Breastfeeding (*n* = 326)			
A mother instinctively knows how to breastfeed	37 (11.4%)	58 (17.8%)	231 (70.9%)
Breastfeeding supports mother–infant bonding	309 (94.8%)	12 (3.7%)	5 (1.5%)
Breastfeeding is more convenient than formula feeding	258 (79.1%)	55 (16.9%)	13 (4.0%)
If a mother is struggling to breastfeed, she should be encouraged to use formula	21 (6.4%)	109 (33.4%)	196 (60.1%)
Infant formula is as healthy for an infant as breastmilk	22 (6.7%)	39 (12.0%)	265 (81.3%)
Formula feeding is the better choice if the mother plans to go out to work	12 (3.7%)	49 (15.0%)	265 (81.3%)
The clinician should assist the mother to make a fully informed and appropriate decision about infant feeding	310 (95.1%)	14 (4.3%)	2 (0.6%)
Breastfeeding beyond 12 months has no health benefits to the infant	35 (10.7%)	62 (19.0%)	229 (70.3%)
I think breastfeeding problems are the domain of a midwife or lactation consultant, not a doctor	37 (11.4%)	61 (18.7%)	228 (69.9%)

Overall knowledge was good with a mean score of 9.0/12 (75%). However, knowledge about discarding breast milk after radiological procedures and antibiotic side effects was low (correct responses 45% and 20% respectively) (Table S1).

The overall mean confidence score was 4.8/7 (68.6%). Most respondents (87%) said that they personally supported women in initiating and maintaining breastfeeding and that they helped facilitate skin‐toto‐skin contact between newborn infants and mothers at all births (89%). Only one in three respondents (37%) stated that they felt confident in managing breastfeeding challenges, and around half (55%) said that they could tell if an infant was sucking well at the breast (Table 2).

Those who sought extra training and had personally breastfed were more likely to have higher attitude scores (*p* = 0.02 and *p* = 0.003 respectively) (Table 3). These two factors remained significant even after adjusting for sex, role, extra training and breastfeeding history: Extra training (β = 0.44 (95% CI 0.04, 0.84) *p* = 0.03) and personal breastfeeding (β = 0.37 (95% CI 0.15, 0.60) *p* = 0.001) (Table S2).

**TABLE 3:** Attitudes, knowledge and confidence scores for practitioners providing breastfeeding (BF) support and care by demographic characteristics.

		Attitudes and behaviours				Knowledge				Confidence			
*N*		Mean (SD)	% correct (SD^1^)	MD^2^ (95% CI^3^)	*p*	Mean (SD)	% correct (SD)	MD (95% CI)	*p*	Mean (SD)	% correct (SD)	MD (95% CI)	*p*
Sex													
Male	66	6.20 (1.69)	68.89 (18.8)	−0.34 (−0.74, 0.07)	0.10	7.89 (1.88)	65.75 (15.7)	−1.48 (−1.92, −1.04)	< 0.001	4.53 (1.54)	64.71 (22.0)	−0.30 (−0.71, 0.10)	0.14
Female	241	6.53 (1.40)	72.56 (15.5)			9.37 (1.54)	78.08 (12.8)			4.83 (1.45)	69.00 (20.7)		
Age (years)													
20–39	109	6.50 (1.49)	72.22 (16.5)		0.80	9.63 (1.39)	80.25 (11.6)		< 0.001	4.56 (1.30)	65.14 (18.6)		0.02
40–59	147	6.37 (1.45)	70.78 (16.1)			8.95 (1.70)	74.58 (14.2)			4.73 (1.53)	67.57 (21.9)		
≥ 60	46	6.43 (1.66)	71.44 (18.4)			8.26 (1.96)	68.83 (16.3)			5.3 (1.56)	75.71 (22.3)		
Country of training													
Australia/NZ	260	6.42 (1.48)	71.33 (16.4)	−0.14 (−0.58, 0.31)	0.55	9.15 (1.70)	76.25 (14.2)	0.69 (0.18, 1.20)	0.01	4.79 (1.45)	68.43 (20.7)	0.12 (−0.32, 0.56)	0.59
Other	52	6.56 (1.58)	72.89 (17.5)			8.46 (1.80)	70.50 (15.0)			4.67 (1.59)	66.71 (22.8)		
Role													
Specialist	233	6.42 (1.53)	71.33 (17.0)	−0.08 (−0.46, 0.30)	0.68	8.83 (1.80)	73.58 (15.0)	−0.82 (−1.25, −0.38)	< 0.001	4.89 (1.50)	69.86 (21.4)	0.19 (0.08, 0.83)	0.02
Trainee	79	6.51 (1.38)	72.33 (15.3)			9.65 (1.37)	80.42 (11.4)			4.43 (1.35)	63.29 (19.2)		
Specific role													
Gynaecology (Gyn)	21	6.95 (1.53)	77.22 (17.0)		0.19	9.05 (2.25)	75.42 (18.8)		0.41	4.81 (1.86)	68.71 (26.6)		0.97
Obstetrics (Obs)	66	6.26 (1.43)	69.56 (15.9)			9.35 (1.43)	77.92 (11.9)			4.80 (1.44)	68.57 (20.6)		
Obs and Gyn	216	6.48 (1.50)	72.00 (16.7)			8.94 (1.77)	74.50 (14.8)			4.77 (1.43)	68.14 (20.4)		
Other	9	5.89 (1.36)	65.44 (15.1)			9.11 (1.54)	75.92 (12.8)			4.56 (1.94)	65.14 (27.7)		
Formal BF training													
Yes	150	6.51 (1.49)	72.33 (16.6)	0.12 (−0.21, 0.46)	0.48	9.17 (1.65)	76.42 (13.8)	0.26 (−0.13, 0.65)	0.19	4.83 (1.41)	69.00 (20.1)	0.10 (−0.24, 0.43)	0.56
No	155	6.39 (1.48)	71.00 (16.5)			8.92 (1.80)	74.33 (15.0)			4.74 (1.55)	67.71 (22.1)		
Sought extra BF training													
Yes	66	6.82 (1.47)	75.78 (16.3)	0.47 (0.69, 0.88)	0.02	9.14 (1.80)	76.17 (15.0)	0.13 (−0.35, 0.60)	0.59	5.47 (1.33)	78.14 (19.0)	0.88 (0.50, 1.27)	< 0.001
No	246	6.35 (1.48)	70.56 (16.4)			9.01 (1.72)	75.08 (14.3)			4.59 (1.45)	65.57 (20.7)		
BF History													
Personal	193	6.69 (1.37)	74.33 (15.2)		0.003	9.48 (1.49)	79.00 (12.4)		< 0.001	5.07 (1.31)	72.43 (18.7)		< 0.001
Partner	57	6.12 (6.12)	68.00 (19.4)			7.91 (1.94)	65.92 (16.2)			4.68 (1.50)	66.86 (21.4)		
None	55	6.05 (1.46)	67.22 (16.2)			8.76 (1.60)	73.00 (13.3)			3.89 (1.56)	55.57 (22.3)		
Accredited BHFI													
Yes	257	6.48 (1.47)	72.00 (16.3)	0.23 (−0.45, 0.91)	0.51	9.08 (1.79)	75.67 (14.9)	0.38 (−0.42, 1.19)	0.35	4.86 (1.43)	69.43 (20.4)	−0.39 (−1.05, 0.27)	0.24
No	20	6.25 (1.65)	69.44 (18.3)			8.7 (1.46)	72.50 (12.1)			5.25 (1.65)	75.00 (23.6)		

Abbreviation: BHFI, baby friendly hospital initiative.

^1^ Standard Deviation.

^2^ Mean Difference based on original mean scores only presented for two‐group comparisons.

^3^ Confidence Interval.

Participants with higher knowledge scores were more likely to be female, younger in age, trainees and those with a personal breastfeeding history (*p* < 0.001) (Table 3). After adjusting for factors of interest, knowledge scores remained higher for females (β = 0.58 (95% CI 0.10, 1.07) *p* = 0.02) and those who had personally breastfed (β = 0.45 (95% CI 0.21, 0.69) *p* < 0.001).

Compared with trainees, specialists had higher scores in confidence (4.9/7 vs. 4.4/7, *p* = 0.02) despite having lower overall scores in knowledge (8.8/12 vs. 9.7/12, p = < 0.001) (Table 3). After adjusting for sex, age, role, extra training and breastfeeding history, increased age (β = 0.39 (95% CI 0.64, 0.71) *p* = 0.002), extra training (β = 0.84 (95% CI 0.46, 1.21) *p* < 0.001) and personal breastfeeding (β = 0.52 (95% CI 0.31, 0.73) *p* < 0.001) were the most important factors associated with increased confidence (Table S2). Participants with a personal breastfeeding history scored higher in all three sections when compared with those without. Those who stated that they would value further education were more likely to be less than 40 years old, still in training, and not have formal breastfeeding training (All p = < 0.01) (Table 3).

### 3.1 Qualitative Results

**FIGURE 1:**
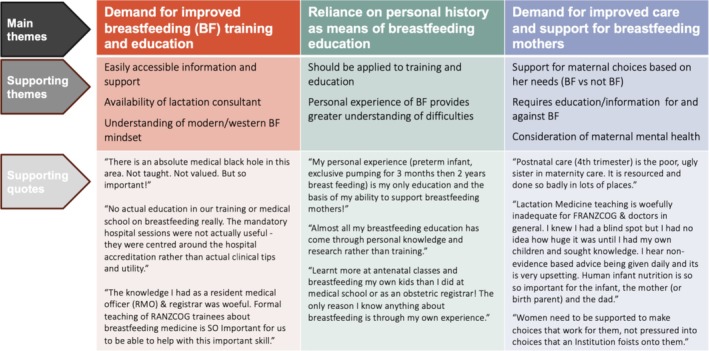
Free‐text comments by respondents classified by theme.

An optional comment was completed by 13% of respondents (*n* = 39), the majority (77%) of whom were FRANZCOG/specialists (Figure 1). Comments were grouped into the following themes: (1) demand for improved breastfeeding training and education, (2) reliance on personal history as a means of breastfeeding education and (3) demand for improved care and support for breastfeeding mothers. The majority of comments were about reliance on personal history as a means of breastfeeding education and were strongly encouraging of the need for further breastfeeding education and training.

## 4 Discussion

To our knowledge, this is the first study to investigate the breastfeeding knowledge, confidence and attitudes among O&G trainees and specialists in Australia and New Zealand. This study found that many participants lacked confidence about breastfeeding and that the majority (60%) of doctors in O&G wanted more formal and improved education and training. Although knowledge was generally high, there were some areas of inadequate breastfeeding knowledge.

The positive attitude scores in this study were similar to previous studies investigating other groups of healthcare professionals [14, 22]. However, the knowledge section in this study's survey was more heterogenous. The questions specifically relevant to O&G doctors, such as maternal antibiotic side effects, breastfeeding restrictions post radiological contrast, appropriate advice for women with low milk supply and choice of antibiotics for mastitis, were less likely to be answered correctly. Findings from previous research investigating general practitioners in Australia and the UK suggest that the doctor's level of knowledge had a greater impact on their confidence than their attitudes towards breastfeeding [22, 23]. However, this study showed that the doctors' level of training had a greater impact on their confidence than their level of knowledge.

Similar to previous studies, we found personal breastfeeding history was the greatest predictor of higher confidence and, as demonstrated in the free‐text comments, it was highlighted as the most useful and beneficial source of breastfeeding education [16, 23, 24]. Those with a personal breastfeeding history also demonstrated higher knowledge and attitude scores. This is similar to a study by Brodribb et al., who found more positive breastfeeding attitudes and higher knowledge scores in doctors with a substantial breastfeeding history, which they defined as having more than 26‐week cumulative personal breastfeeding experience (self or partner) [22, 25, 26]. Personal experience, however, is not an adequate nor appropriate resource to rely on in providing evidence‐based information and support to patients.

This study's findings are similar to other Australian and international studies [16, 26, 27] investigating the breastfeeding knowledge and confidence among other health professionals, including breast surgeons, paediatricians and general practitioners. These studies report that the majority of doctors do not think they have adequate breastfeeding education and would value further training. In our study, participants wanting more breastfeeding education were more likely to be younger, trainees and those who did not have formal breastfeeding education. Inadequate knowledge may contribute to limited support and suboptimal management of breastfeeding mothers. A previous study by Finneran and Murphy [28] found that general practitioners who had formal training in breastfeeding were more likely to promote it to their patients. Research shows that improving physician education in breastfeeding improves maternal and newborn breastfeeding outcomes [18, 19]. This study suggests a potential need for the development of educational resources uniquely designed for O&G specialists and trainees.

Strengths of this study included the survey, including specialists and trainees from a wide geographical area of two countries. Given the high percentage of respondents with a breastfeeding history (81%), the participants included in this study may have had a greater interest in breastfeeding than nonrespondents. Alternatively, as suggested by the free‐text comments, participants with a personal breastfeeding history might have valued the importance of improving breastfeeding education, given their own lack of support or difficulties in breastfeeding as mothers and/or partners of breastfeeding mothers. Nonetheless, findings indicating higher knowledge scores among participants with a personal breastfeeding history suggest that even lower knowledge levels might prevail among nonrespondents who chose not to engage with this study. Limitations of the study include the low response rate (11%), which was not dissimilar to other surveys conducted among members of the RANZCOG [29] or an Australian study surveying breastfeeding knowledge among GPs [22]. The higher percentage of female respondents in this study is representative of the gender ratio within the college, with 83% of trainees being female [21]. Another limitation of this study was the small number of knowledge questions, which was thought to be necessary to make the study feasible for distribution and completion. A larger knowledge section may have produced a more comprehensive reflection of participant knowledge strengths and deficits. Quantifying what formal breastfeeding training participants have had would have yielded a more comprehensive understanding of the background education and training of this study's participants.

Overall confidence about breastfeeding in RANZCOG specialists and trainees was moderate, with poor confidence in managing breastfeeding challenges. The majority of respondents wanted more breastfeeding education and training. Breastfeeding is a critical public health matter with maternal and infant benefits and long‐term impacts. Given the low breastfeeding rates in Australia and New Zealand, addressing strategies to improve breastfeeding support is an important issue that could have a direct impact on maternal and infant care. The findings from this study can be utilised to develop and inform educational programs and guidelines, specifically catered towards O&G doctors and trainees to improve knowledge and confidence about breastfeeding and its impact on women's and infants' health. Regular training and updates should be implemented as part of ongoing clinical practice guidelines.

## Conflicts of Interest

The authors declare no conflicts of interest.

## Supporting information


**Data S1.** Supporting Information.

## References

[1] A. Brown , “Breastfeeding as a Public Health Responsibility: A Review of the Evidence,” Journal of Human Nutrition and Dietetics 30, no. 6 (2017): 759–770. 28744924 10.1111/jhn.12496

[2] American College of Obstetricians and Gynecologists (ACOG) , “Optimizing Support for Breastfeeding as Part of Obstetric Practice. Committee Opinion No. 658,” (2016).

[3] Centers for Disease Control and Prevention , “Breastfeeding: Why it Matters,” (2023), https://www.cdc.gov/breastfeeding/about‐breastfeeding/why‐it‐matters.html.

[4] J. Willumsen , “Breastfeeding Education for Increased Breastfeeding Duration,” (2013), https://www.who.int/elena/bbc/breastfeeding_education/en/.

[5] Australian Institute of Health and Welfare, (AIHW) , “2010 Australian National Infant Feeding Survey: Indicator Results,” (2012).

[6] NHMRC (National Health and Medical Research Council) , “Infant Feeding Guidelines: Information for Health Workers, NHMRC, Australian Government,” (2015).

[7] Baby Friendly Hospital Initiative (BFHI) Australia , BFHI Handbook for Maternity Facilities (BFHI, Australia, 2020).

[8] W. Brodribb , T. Fallon , C. Jackson , and D. Hegney , “Attitudes to Infant Feeding Decision‐Making–a Mixed‐Methods Study of Australian Medical Students and GP Registrars,” Breastfeeding Review 18, no. 1 (2010): 5–13. 20443434

[9] A. Gavine , S. MacGillivray , M. J. Renfrew , L. Siebelt , H. Haggi , and A. McFadden , “Education and Training of Healthcare Staff in the Knowledge, Attitudes and Skills Needed to Work Effectively With Breastfeeding Women: A Systematic Review,” International Breastfeeding Journal 12, no. 1 (2016): 6. 28167998 10.1186/s13006-016-0097-2 PMC5288894

[10] K. V. Biggs , K. J. Fidler , N. S. Shenker , and H. Brown , “Are the Doctors of the Future Ready to Support Breastfeeding? A Cross‐Sectional Study in the UK,” International Breastfeeding Journal 15 (2020): 1–8. 32434558 10.1186/s13006-020-00290-z PMC7238622

[11] A. Gavine , S. C. Shinwell , P. Buchanan , et al., “Support for Healthy Breastfeeding Mothers With Healthy Term Babies,” Cochrane Database of Systematic Reviews 28, no. 2 (2022): CD001141. 10.1002/14651858.CD001141.pub6 PMC9595242 36282618

[12] E. Esselmont , K. Moreau , M. Aglipay , and C. M. Pound , “Residents' Breastfeeding Knowledge, Comfort, Practices, and Perceptions: Results of the Breastfeeding Resident Education Study (BRESt),” BMC Pediatrics 18, no. 1 (2018): 1–7. 29788928 10.1186/s12887-018-1150-7 PMC5964719

[13] A. M. Sims , S. A. Long , J. A. Tender , and M. A. Young , “Surveying the Knowledge, Attitudes, and Practices of District of Columbia ACOG Members Related to Breastfeeding,” Breastfeeding Medicine 10, no. 1 (2015): 63–68. 25389912 10.1089/bfm.2014.0066

[14] C. M. Pound , K. Williams , R. Grenon , M. Aglipay , and A. C. Plint , “Breastfeeding Knowledge, Confidence, Beliefs, and Attitudes of Canadian Physicians,” Journal of Human Lactation 30, no. 3 (2014): 298–309. 24919510 10.1177/0890334414535507

[15] O. Holtzman and T. Usherwood , “Australian General Practitioners' Knowledge, Attitudes and Practices Towards Breastfeeding,” PLoS One 13, no. 2 (2018): e0191854. 29489841 10.1371/journal.pone.0191854 PMC5830034

[16] W. Brodribb , A. B. Fallon , C. Jackson , and D. Hegney , “Breastfeeding Knowledge: The Experiences of Australian General Practice Registrars,” Australian Family Physician 38, no. 1/2 (2009): 26–29. 19283232

[17] US Preventive Services Task Force , “Primary Care Interventions to Support Breastfeeding: US Preventive Services Task Force Recommendation Statement,” Jama 316, no. 16 (2016): 1688–1693. 27784102 10.1001/jama.2016.14697

[18] A. V. Holmes , A. Y. McLeod , C. Thesing , S. Kramer , and C. R. Howard , “Physician Breastfeeding Education Leads to Practice Changes and Improved Clinical Outcomes,” Breastfeeding Medicine 7, no. 6 (2012): 403–408. 23046226 10.1089/bfm.2012.0028

[19] A. M. DiGirolamo , L. M. Grummer‐Strawn , and S. B. Fein , “Do Perceived Attitudes of Physicians and Hospital Staff Affect Breastfeeding Decisions?,” Birth 30, no. 2 (2003): 94–100. 12752166 10.1046/j.1523-536x.2003.00227.x

[20] P. A. Harris , B. L. Taylor , V. Minor , et al., “The REDCap Consortium: Building an International Community of Software Partners,” Journal of Biomedical Informatics (2019), 10.1016/j.jbi.2019.103208. PMC7254481 31078660

[21] The Royal Australian and New Zealand College of Obstetricians and Gynaecologists (RANZCOG) , “RANZCOG Activities Report 2020‐2021,” (2021).

[22] W. Brodribb , A. Fallon , C. Jackson , and D. Hegney , “Breastfeeding and Australian GP Registrars—Their Knowledge and Attitudes,” Journal of Human Lactation 24, no. 4 (2008): 422–430. 18974291 10.1177/0890334408323547

[23] J. Ingram , “Multiprofessional Training for Breastfeeding Management in Primary Care in the UK,” International Breastfeeding Journal 1 (2006): 1–7. 16722540 10.1186/1746-4358-1-9 PMC1475559

[24] S. Nakar , O. Peretz , R. Hoffman , Z. Grossman , B. Kaplan , and S. Vinker , “Attitudes and Knowledge on Breastfeeding Among Paediatricians, Family Physicians, and Gynaecologists in Israel,” Acta Paediatrica 96 (2007): 848–851. 17537013 10.1111/j.1651-2227.2007.00310.x

[25] M. L. Power , E. Locke , J. Chapin , L. Klein , and J. Schulkin , “The Effort to Increase Breast‐Feeding. Do Obstetricians, in the Forefront, Need Help?,” Journal of Reproductive Medicine 48, no. 2 (2003): 72–78. 12621789

[26] R. J. Schanler , K. G. O'Connor , and R. A. Lawrence , “Pediatricians' Practices and Attitudes Regarding Breastfeeding Promotion,” Pediatrics 103, no. 3 (1999): e35. 10049991 10.1542/peds.103.3.e35

[27] H. M. Johnson , M. Teshome , P. Singh , and K. B. Mitchell , “Lactation Education for Surgeons: American Society of Breast Surgeons (ASBrS) Survey Demonstrates Strong Member Interest in Expanded Training,” Annals of Surgical Oncology 30, no. 10 (2023): 6125–6132. 37452168 10.1245/s10434-023-13882-w

[28] B. Finneran and K. Murphy , “Breast Is Best for GPs‐Or Is It? Breastfeeding Attitudes and Practice of General Practitioners in the Mid‐West of Ireland,” Irish Medical Journal 97, no. 9 (2004): 268–270. 15568583

[29] A. W. Shand , M. E. Harpham , A. Lainchbury , L. McCormack , S. Leung , and N. Nassar , “Knowledge, Advice and Attitudes Toward Women Driving a Car After Caesarean Section or Hysterectomy: A Survey of Obstetrician/Gynaecologists and Midwives,” Australian & New Zealand Journal of Obstetrics & Gynaecology 56, no. 5 (2016): 460–465. 27396855 10.1111/ajo.12496

