# Supplementary material for: Current Breastfeeding Attitudes, Knowledge and Confidence of Obstetricians and Gynaecologists in Australia and New Zealand
Source: Aust N Z J Obstet Gynaecol. 2025 Jul 22;66(2):e70055. doi: 10.1111/ajo.70055 (PMC12946589; doi:10.1111/ajo.70055)
Supplement: Supplementary file 1 — Data S1. Supporting Information. [file AJO-66-0-s001.docx]

**Supplementary Table 1. Percentage of correctly answered knowledge questions about breastfeeding**

| **Knowledge questions** | **Answered correctly** | |
| --- | --- | --- |
|  | **Number (n=316)** | **%** |
| Milk production maintenance: removal of breastmilk as essential to milk production | 294 | 93.0 |
| Exclusive breastfeeding benefits: exclusive breastfeeding as most beneficial form of infant feeding for first six months | 256 | 81.0 |
| Feeding times in breastfeeding infant: normal breastfeeding routine in the first few weeks of life | 286 | 90.5 |
| Contraindications to breastfeeding: maternal hepatitis C | 257 | 81.3 |
| Low milk supply: appropriate advice for women with low milk supply | 202 | 63.9 |
| Positioning and attachment: most common cause of cracked nipples | 277 | 87.7 |
| Mastitis and continual breastfeeding: recommendations to women with mastitis | 283 | 89.6 |
| Antibiotics for mastitis: drug of choice in treatment of mastitis | 240 | 75.9 |
| Antenatal expression in women with diabetes: safety of antenatal expression and reduction of incidence of hypoglycaemia | 274 | 86.7 |
| Maternal antibiotic side effects to infant: gastrointestinal side effects in breastfeeding babies | 63 | 19.9 |
| Discarding milk after radiology: breastfeeding requirements post radiological contrast | 141 | 44.6 |
|  |  |  |
| Breastfeeding and anaesthesia: safety of continued breastfeeding immediately after anaesthesia | 280 | 88.6 |

*Questions answered correctly classified as ‘strongly agree’, ‘agree’, ‘strongly disagree’ or ‘disagree’. Those who answered ‘neither agree nor disagree’ or ‘don’t know’ were not included.

**Supplementary Table 2: Factors associated with attitude, knowledge and confidence scores**

| **Outcome and main factors of interest** | **Univariate analysis** | **Multivariate analysis*** | |
| --- | --- | --- | --- |
|  | β (95%CI) | β (95%CI) | p-value |
| Attitude |  |  |  |
| Sex (female) | 0.18 (-0.21, 0.57) | -0.16 (-0.57, 0.26) | 0.47 |
| Role (trainee) | 0.08 (-0.30, 0.46) | 0.17 (-0.22, 0.56) | 0.38 |
| Sought extra training | 0.47 (0.07, 0.88) | 0.44 (0.04, 0.84) | 0.03 |
| BF History | 0.35 (0.14, 0.56) | 0.37 (0.15, 0.60) | 0.001 |
| Knowledge |  |  |  |
| Sex (female) | 1.24 (0.82, 1.67) | 0.58 (0.10, 1.07) | 0.02 |
| Age group | -0.69 (-0.96, -0.42) | -0.28 (-0.65, 0.09) | 0.14 |
| Country (Aus/NZ) | 0.69 (0.18, 1.20) | 0.43 (0.06, 0.92) | 0.08 |
| Role (trainee) | 0.82 (0.38, 1.25) | 0.45 (-0.10, 1.00) | 0.11 |
| BF History | 0.53 (0.29, 0.77) | 0.45 (0.21, 0.69) | <.001 |
| Confidence |  |  |  |
| Sex (female) | 0.27 (-0.11, 0.65) | 0.16 (-0.26, 0.59) | 0.39 |
| Age group | 0.33 (0.09, 0.57) | 0.39 (0.64, 0.71) | 0.002 |
| Role (trainee) | -0.46 (-0.83, -0.08) | 0.06 (-0.41, 0.53) | 0.80 |
| Sought extra training | 0.88 (0.5, 01.27) | 0.84 (0.46, 1.21) | <.001 |
| BF History | 0.56 (0.36, 0.76) | 0.52 (0.31, 0.73) | <.001 |

* Adjusted for factors where p-value <0.2 on univariate analyses;

β=Beta coefficient unstandardized, CI- Confidence Interval
